# Bilateral Cystoid Macular Edema After Light Treatment With Light-Adjustable Lens

**DOI:** 10.7759/cureus.90493

**Published:** 2025-08-19

**Authors:** Fatma Shakarchi, Evelyn Bassett, Ayorinde Cooley, Curtis R Martin, Traeson Brandenburg, Isabelle Dien, Lisia B Ferreira, Christopher Shelby, Wyche T Coleman, Stephen LoBue

**Affiliations:** 1 Ophthalmology, Willis-Knighton Medical Center, Shreveport, USA; 2 Ophthalmology, LoBue Laser and Eye Medical Center, Murrieta, USA; 3 Ophthalmology, Centro de Medicina Humanizado, Mexico City, MEX

**Keywords:** case report, cataract, cystoid macular edema, lal, light-adjustable intraocular lenses

## Abstract

Light-adjustable lenses (LALs) allow for visual acuity (VA) adjustment after cataract surgery, using ultraviolet (UV) light therapy. UV-induced cystoid macular edema (CME) is not well documented. We report the first case of bilateral CME after UV light therapy with LAL. An 81-year-old woman underwent uncomplicated staged bilateral cataract extraction with LAL implantation. Two days after the second UV light adjustment, she had bilateral decreased VA and a new-onset bilateral CME. UV treatments were halted, and she was started on topical nonsteroidal anti-inflammatory drug (NSAID) therapy and prednisolone. CME completely resolved within three weeks following onset. Topical NSAID and prednisone drops were continued until the completion of all light treatments, followed by a slow taper over one month. We hypothesize that UV light can contribute to CME via multiple mechanisms, including UV-activated macromer byproducts, direct UV-induced injury, or ultraviolet B (UVB) disrupting the blood-aqueous barrier. Clinicians should be aware of this complication and maintain a low threshold for deferring light therapy and initiating topical treatment.

## Introduction

Light-adjustable lenses (LALs) offer an innovative option in refractive cataract surgery as they allow noninvasive postoperative adjustment to the lens power. Its refractive power is adjusted postoperatively by modifying the silicone macromers through directing ultraviolet (UV) light on the intraocular lens [1]. LALs are considered safe and have good visual outcomes [2,3]. They are composed of photosensitive silicone macromers that polymerize when exposed to UV light. This adjustment process is performed using a light delivery device that emits 365 nm UV light in up to three treatments with two "lock-in" procedures to prevent migration from the refractive target. This technology can be advantageous in patients with a history of refractive surgery or those with complex refractive goals.

In an FDA study of 600 patients, eyes that received LAL were twice as likely to achieve an uncorrected distance visual acuity (UDVA) of 20/20 than those with standard monofocal intraocular lenses (IOLs) [2]. In the FDA study, three eyes (0.7%) had cystoid macular edema (CME), with one requiring sub-Tenon triamcinolone acetonide injection, which caused delay of light treatment [2]. 

The development of CME after cataract surgery occurs in up to one in 400 cataract surgeries [4]. It manifests as fluid accumulation in the retina between the outer plexiform layer and the inner nuclear layer around the fovea and may lead to a decrease in best-corrected visual acuity (BCVA), micropsia, metamorphopsia, and central scotoma [5]. Different factors have been suggested to contribute to the pathogenesis of CME secondary to cataract surgery, such as surgical complications, systemic conditions, age, and medications [6,7]. However, UV-induced CME is not well documented. Thus, we present the first documented case report of bilateral CME after UV light therapy with LAL and its postoperative outcome and management.

## Case presentation

**Figure 1:**
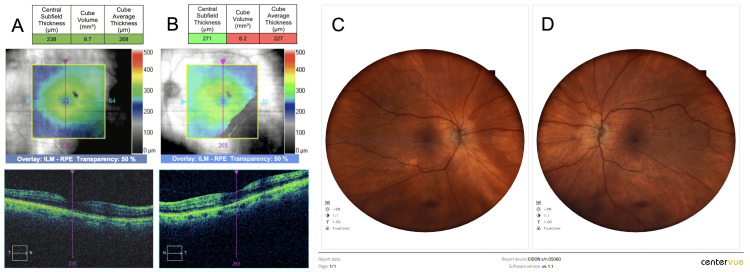
Preoperative spectral domain OCT and fundus image of both eyes (A, B) Preoperative spectral domain OCT of both eyes demonstrates normal retinal architecture with preserved foveal contour, intact ellipsoid zone, and no evidence of cystoid changes or epiretinal membranes. Central subfield thickness measured 238 µm in the right eye and 271 µm in the left eye. (C, D) Corresponding preoperative fundus photographs of both eyes reveal normal macular appearance without signs of retinal pathology. OCT: optical coherence tomography

An 81-year-old woman with a medical history significant for hypertension, hypothyroidism, and high cholesterol levels presented for bilateral cataract extraction. Her ocular history was significant for keratoconjunctivitis sicca and bilateral myopic laser-assisted in situ keratomileusis (LASIK) procedure in 2005. She had no history of diabetes, retinal vascular disease, or uveitis, and she was not on prostaglandin analogues, anticoagulants, or known photosensitizing or retinotoxic agents. Her preoperative visual acuity (VA) was 20/50 in the right eye (OD) and 20/80-1 in the left eye (OS), and spectral domain optical coherence tomography (OCT; Cirrus HD-OCT, Carl Zeiss Meditec, Jena, Germany) showed normal foveal contour, intact ellipsoid zone, and no cystoid changes or epiretinal membranes in both eyes. The central subfield thickness (CST) measured 238 µm OD and 271 µm OS, with macular cube volumes of 9.7 mm³ OD and 8.2 mm³ OS, respectively (Figure 1A-1D).

The patient underwent uncomplicated sequential phacoemulsification cataract extraction with the implantation of LAL (RxSight, Aliso Viejo, California, United States) in the capsular bag, with a two-week interval between surgeries in both eyes. A 23 D LAL and 23.5 D LAL were implanted in the right eye and left eye, respectively. Her immediate postoperative course was unremarkable, and her UDVA improved to 20/25 in both eyes (OU) by postoperative week 1. The standard LAL protocol was initiated one month postoperatively, including UV light treatments for refractive adjustment. OD was targeted for plano and OS for -2.00. After one adjustment, the patient's UDVA was 20/25 OD and uncorrected near-visual acuity (UNVA) 20/25 OS. Manifest refraction was -0.25 OD and -1.75 -0.50×180 OS. A 1+ posterior capsule opacification (PCO) was noted OU. A neodymium-doped yttrium aluminum garnet (Nd:YAG) capsulotomy was performed OU with no complications. UDVA and UNVA improved to 20/20 OD and 20/20 OS. The patient was satisfied with their uncorrected visual acuity, electing to lock in their vision bilaterally.

**Figure 2:**
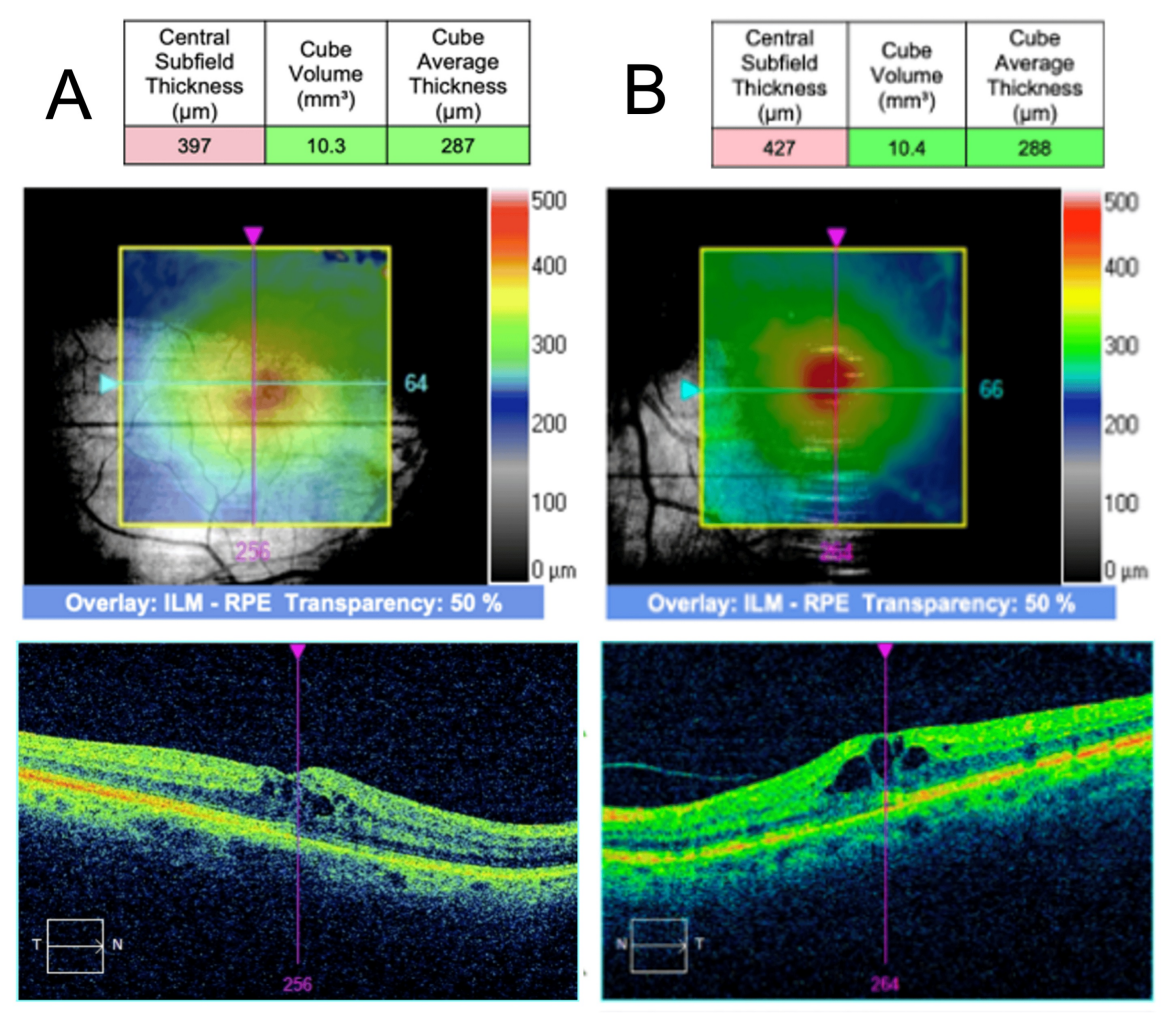
Bilateral CME one week following UV treatment SD-OCT one week following the second UV light treatment, demonstrating CME in both eyes. (A) The right eye demonstrates increased CST of 397 µm. (B) The left eye demonstrates increased CST of 427 µm. (A-B) Intraretinal cystic spaces were localized primarily within the inner nuclear layer. No epiretinal membranes or vitreomacular interface abnormalities were present in either eye. CME: cystic macular edema; UV: ultraviolet; SD-OCT: spectral domain optical coherence tomography; CST: central subfield thickness

The first series of UV lock-in treatments was completed uneventfully on January 29th. Within one week after the first UV light lock-in, the patient noticed a decrease in her uncorrected VA to 20/40 for distance and near. A repeat OCT revealed a new-onset bilateral CME with intraretinal cystic changes OU, with CST at 397 µm OD and 427 µm OS (Figure 2). No concomitant epiretinal membrane, vitreomacular traction, or subretinal fluid was noted. 

**Figure 3:**
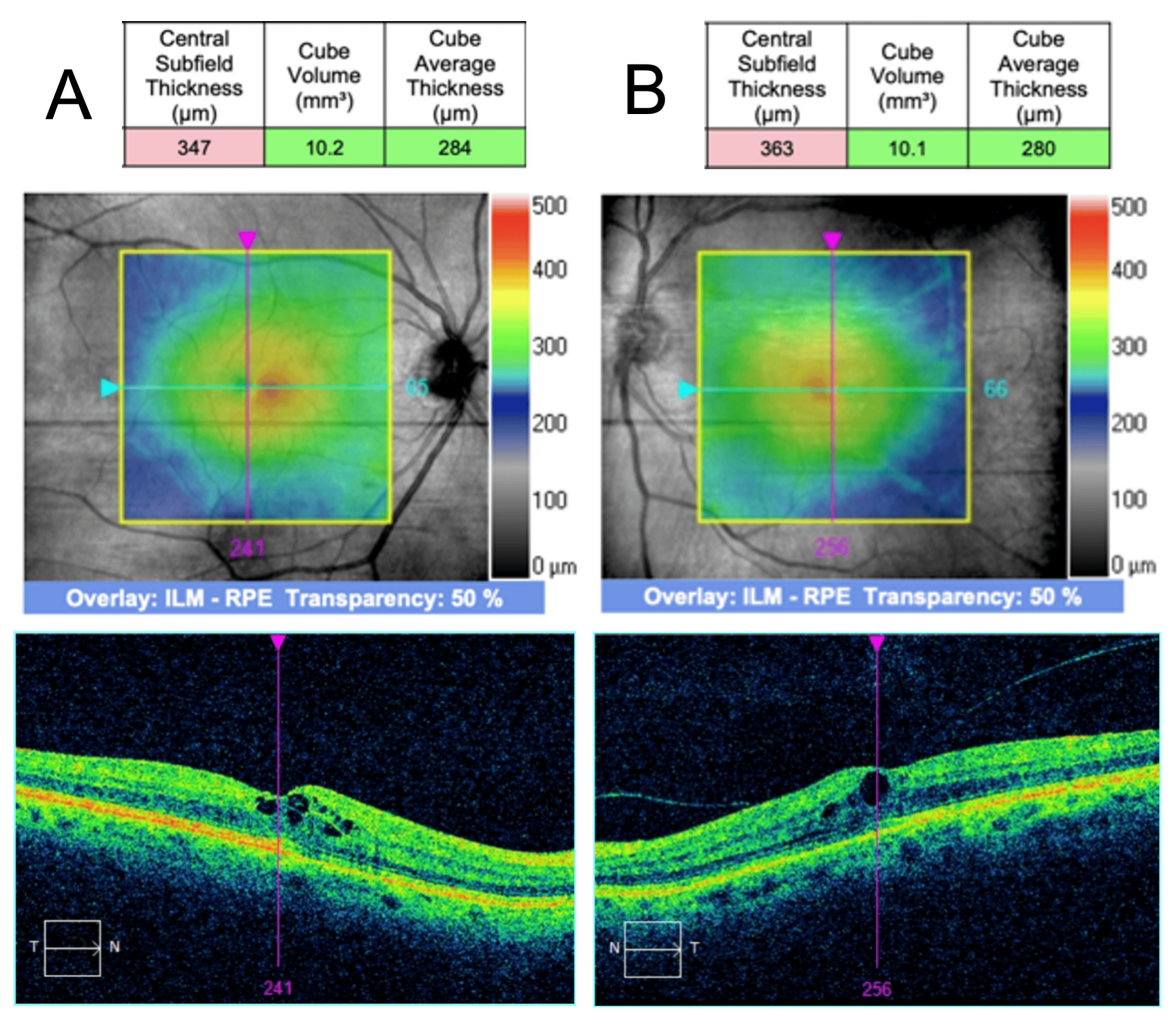
Bilateral CME two weeks following UV treatment SD-OCT findings on follow-up two weeks after CME onset. (A) The CST improved to 347 µm in the right eye and (B) 363 µm in the left eye. CME: cystic macular edema; UV: ultraviolet; SD-OCT: spectral domain optical coherence tomography; CST: central subfield thickness

**Figure 4:**
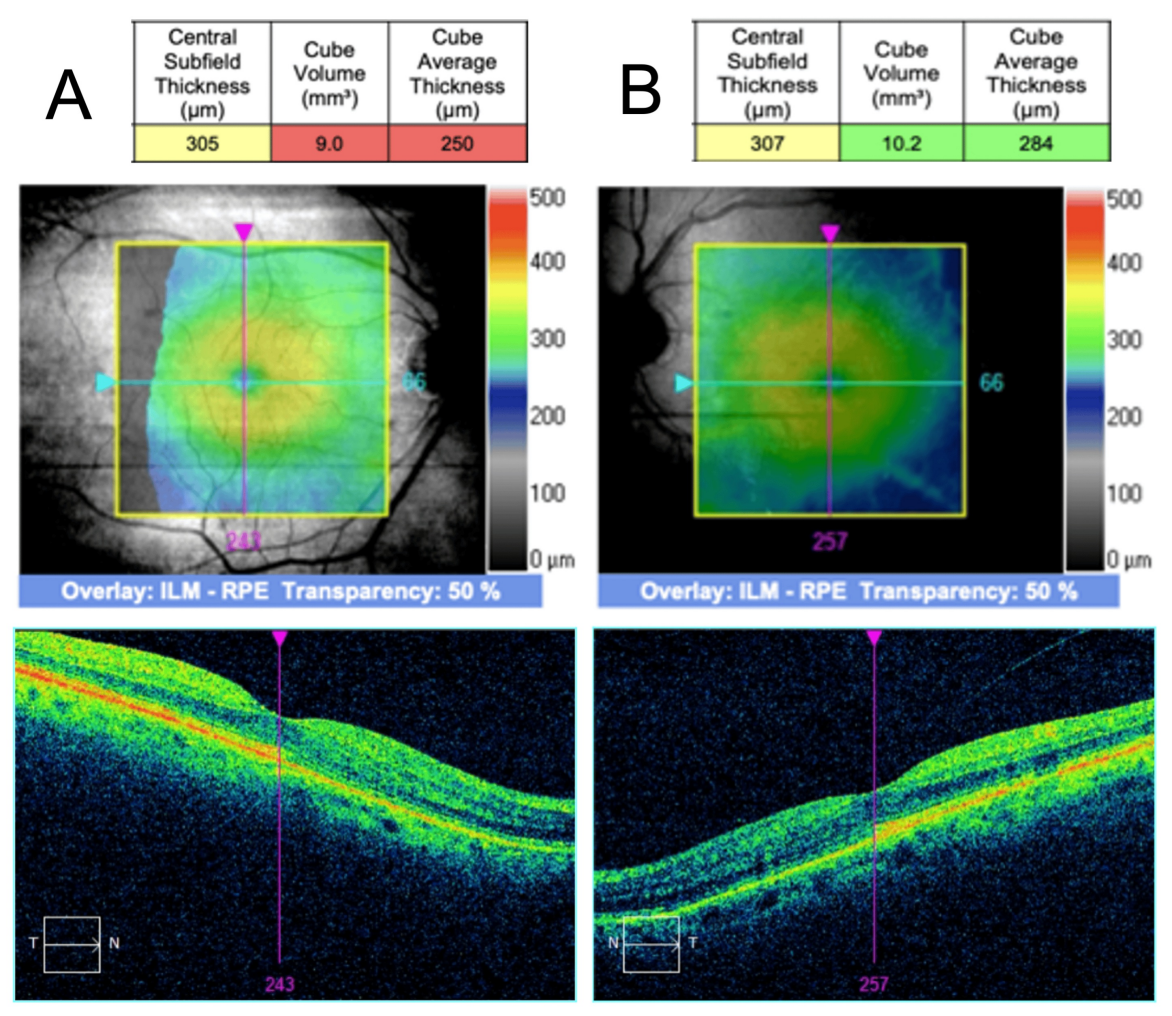
Bilateral CME three weeks following UV treatment SD-OCT findings on follow-up three weeks after CME onset. (A) The CST improved to 305 µm in the right eye and (B) 307 µm in the left eye. (A-B) A normal macular architecture was noted in both eyes with the complete resolution of CME. CME: cystic macular edema; UV: ultraviolet; SD-OCT: spectral domain optical coherence tomography; CST: central subfield thickness

Further UV light therapy was postponed. The patient was started on 1% prednisolone acetate four times daily (QID) and a topical nonsteroidal anti-inflammatory drug (NSAID) therapy 0.5% ketorolac tromethamine (QID). Serial OCT was employed to monitor treatment response. Two weeks after CME onset, CST had decreased to 347 µm OD and 363 µm OS, with minor improvement of intraretinal cysts (Figure 3). At three weeks from the CME onset, CST further improved to 305 µm OD and 307 µm OS with the complete resolution of macular edema (Figure 4). 

**Figure 5:**
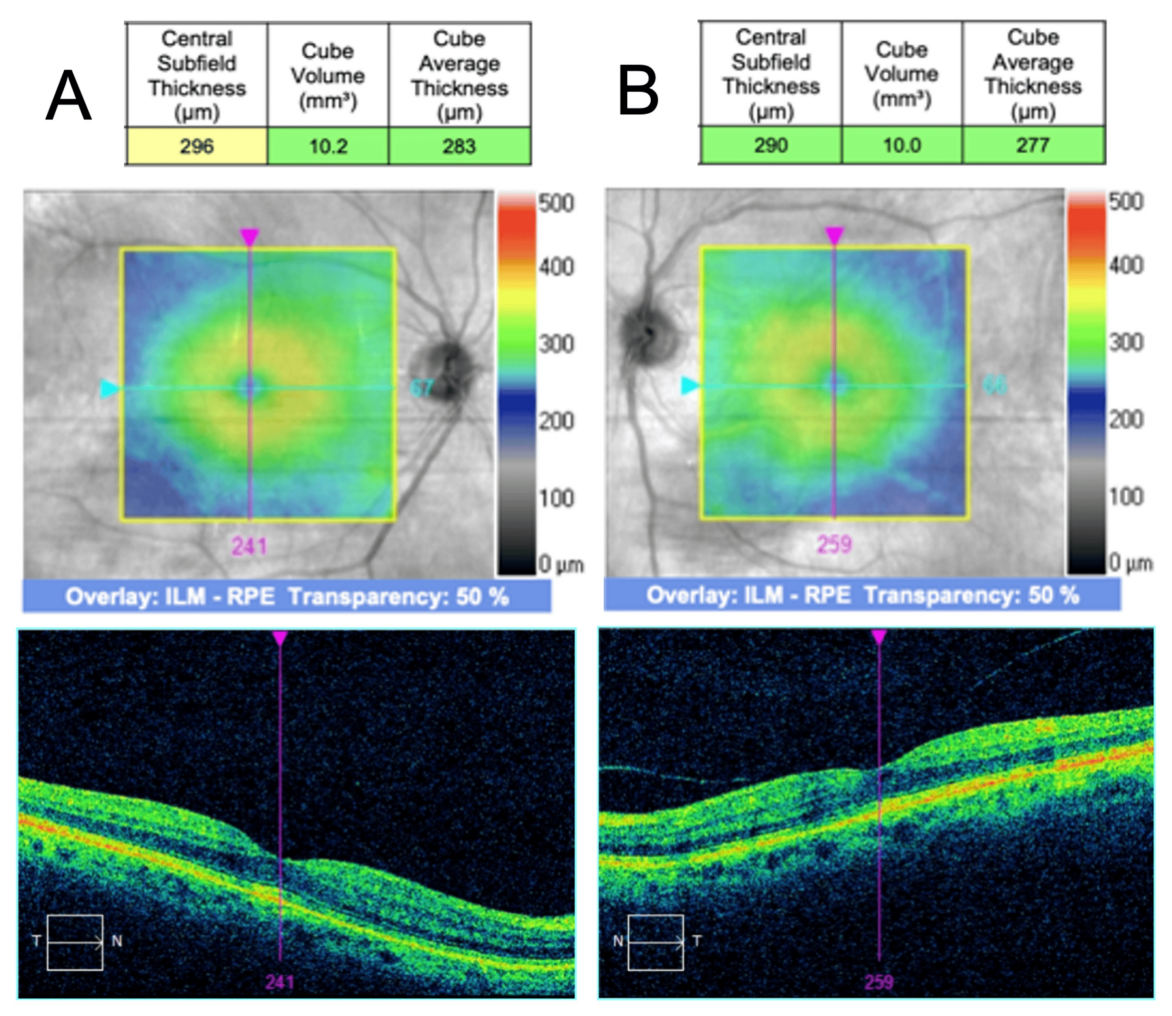
Bilateral CME 10 weeks following UV treatment SD-OCT findings on follow-up 10 weeks after CME onset. (A-B) A normal macular architecture was documented in both eyes with the complete resolution of CME after completing stopping all topical drops one month prior. CME: cystic macular edema; UV: ultraviolet; SD-OCT: spectral domain optical coherence tomography; CST: central subfield thickness

One additional UV lock-in treatment was then performed, per standard LAL protocol, while continuing 1% prednisolone acetate QID and a topical 0.5% ketorolac tromethamine QID. The 1% prednisolone acetate and a topical 0.5% ketorolac tromethamine were slowly tapered over the course of a month. Follow-up visits 10 weeks after UV lock-ins revealed no recurrence of macular edema (Figure 5). At this time, the patient's final unaided VA was 20/20 OD and J1+ OS. The chronological order of the patient's progression and resolution of CME is further clarified (Table 1).

**Table 1:** Chronological overview of the patient's course following bilateral LAL implantation and UV treatment The timeline highlights the rapid onset, bilateral symmetry, and favorable response to conservative therapy. OCT: optical coherence tomography; OU: both eyes; CST: central subfield thickness; OD: right eye; OS: left eye; LAL: light-adjustable lens; UV: ultraviolet; Nd:YAG: neodymium-doped yttrium aluminum garnet; CME: cystoid macular edema; QID: four times daily

Date	Event/visit	Key findings/notes
November 18, 2024	Preoperative evaluation	OCT: normal macular architecture OU; CST: 238 µm OD and 271 µm OS
December 9, 2024	Bilateral LAL implantation	LALs implanted OU. Immediate post-op course unremarkable
January 13, 2024	First bilateral UV adjustment	OD target was plano; OS target was -2.00
January 20, 2025	Bilateral Nd:YAG capsulotomy	Completed without complication
January 29, 2025	First UV lock-in	Completed without complication
February 5, 2025	One-week follow-up	Decreased vision OU. OCT: CST: 397 µm OD and 427 µm OS. Intraretinal cystic changes (bilateral CME). UV treatments suspended; initiated prednisolone acetate 1% QID+ketorolac 0.5% QID
February 11, 2025	Two-week follow-up	OCT: CST: 347 µm OD and 363 µm OS; resolution of central cysts; partial symptom improvement
February 18, 2025	Three-week follow-up	OCT: CST: 296 µm OD and 290 OS. Anatomic resolution (no CME)
February 25, 2025	Second UV lock-in	Completed without complication
April 15, 2025	Follow-up	OCT: CST: 296 µm OD and 285 µm OS; complete anatomical and functional recovery

## Discussion

CME is a known postoperative complication of cataract surgery, most commonly associated with surgical trauma and inflammation-induced breakdown of the blood-retinal barrier (BRB) [8]. Typically, CME ensues several weeks post-surgery and is generally unilateral, with an estimated incidence following standard cataract surgery between 0.1% and 2%, depending on risk profile [9]. In the pivotal FDA clinical trial for the LAL [2], CME occurred in 0.7% (3/600) of treated eyes, though detailed reports of laterality and clinical course were not provided. The results of the FDA trial implied that the occurrence of CME was associated with the context of cataract surgery, not a direct effect of UV light treatment.

The development of CME following UV adjustment of LALs may involve multiple mechanisms. UV-activated macromers may release byproducts that trigger low-grade intraocular inflammation, leading to BRB disruption through cytokines, vascular endothelial growth factors, and prostaglandin-mediated vascular permeability. It can involve both the inner BRB (formed by retinal capillary endothelial cells) and the outer BRB (maintained by the retinal pigment epithelium (RPE)). Additionally, direct UV-induced subclinical injury to the retina may contribute to BRB breakdown. During LAL treatment, UV light is typically delivered at 365-370 nm, with lens transmission cut-off at 10% T around 385±2 nm across all lens powers. Treatment duration ranges from 40 to 120 seconds, usually over 1-3 sessions [2]. Although a portion of ultraviolet A (UVA) can reach the retina, most UV phototoxicity is attributed to ultraviolet B (UVB). UVB promotes the disruption of the blood-aqueous barrier and induces anterior segment inflammation in mice [10] and is also known to damage the RPE cells and contribute to age-related macular degeneration [11]. Most experimental research on UVA-induced retinal cytotoxicity has been centered on RPE cells, particularly the ARPE-19 cell line. Findings show that UVA exposure, including the wavelength used for UV light treatments, leads to a significant decrease in RPE barrier impedance, increased oxidative stress, mitochondrial dysfunction, and cell death. Both apoptotic and necrotic pathways appear to be involved, especially with prolonged exposure [12-14]. Similarly, photochemical stress on the Müller glia by UV irradiation may promote local cytokine release, compromising the inner BRB [15].

While these mechanisms remain speculative, the bilateral presentation shortly after synchronized light treatments, along with spontaneous resolution under topical therapy alone, makes this clinical case worth documenting. This possible correlation is supported by the findings of Kraff et al. [16] in a randomized trial of 301 patients receiving IOLs either with or without UV-filtering, where eyes with UV-filtering IOLs had a statistically significant lower incidence of CME (18.8% vs. 9.5%; p=0.03). Similarly, Rosenberg et al. [17] in a randomized trial showed that lower intraoperative light intensity was associated with significantly lower rates of CME postoperatively.

Our findings also contrast with the classic presentation of Irvine-Gass syndrome. First, the onset of CME here reported was accelerated, manifesting within a week of UV exposure. This compressed timeline supports the idea of a unique inflammatory insult linked to the photochemical activation phase rather than the surgical act itself. Second, our case differed in that both eyes developed CME simultaneously and synchronously rather than sequentially, reinforcing the hypothesis of UV-induced bilateral inflammatory insult rather than an independent surgical trauma. A large database study [18] that included 54,209 patients found a relative risk of 8.55 for developing fellow-eye pseudophakic CME following first-eye CME in standard cataract surgery, but this was largely in patients with additional risk factors, including epiretinal membrane, history of retinal vascular disease, and diabetes. None of those were present in this case. Third, there was no vitreoretinal traction or clinically active posterior segment inflammation, and the full anatomical resolution and restoration of VA with topical NSAID and steroid therapy support an inflammatory etiology rather than mechanical or ischemic causes that typically necessitate longer treatment courses and, in some cases, surgical intervention. Of note, a review of the patient's medications revealed no known retinotoxic agents, such as tamoxifen, or photosensitizing agents, such as tetracycline, psoralens, and amiodarone.

Our case also contrasts with several reported cases of bilateral pseudophakic CME in the literature. Tsaousis and Tsokolas [19] reported a case of bilateral and symmetric pseudophakic CME in an 80-year-old woman with macular telangiectasia type 2 following uneventful subsequent cataract surgeries. She responded well to topical treatment (combination of steroids and NSAIDs for the right eye and NSAIDs only for the left eye), and the CME resolved after several months. In our case report, the patient had a quicker resolution (three weeks after CME onset), with no underlying retinal conditions that might have posed an additional risk factor. Moreover, Scarpa [20] reported the case of a 72-year-old woman who developed bilateral CME after an uneventful phacoemulsification of cataract. Conventional medical therapy was unsuccessful, and vitreomacular traction was documented, therefore requiring bilateral vitrectomy surgery, which was associated with improved VA, whereas our patient improved with conservative treatment and had no tractional component. 

Moreover, YAG capsulotomy could contribute to CME development. Allphin et al. recently reported a case where CME developed months after YAG laser vitreolysis [21]. Similarly, Steinert et al. found in a case series that the risk of developing CME following YAG capsulotomy was 1.23% (95% confidence interval: 0.51% to 1.95%), with the timing typically occurring many months after the procedure [22]. However, in this case, the bilateral capsulotomies were completed before the lock-in phase and without immediate postoperative complications. Given the symmetry, timing, and low total YAG energy used (2.4 mJ×10 per eye), CME is more likely attributable to UV-induced inflammatory stress than delayed YAG-associated changes.

To date, no published studies have documented bilateral CME in the context of LAL treatment. The absence of this finding in controlled environments may suggest that this may be an underrecognized phenomenon, particularly if patients present with mild symptoms. Given the increasing use of LALs in refractive cataract surgery, our case report highlights the need for heightened surveillance and raises the question of whether routine OCT screening should be performed prior to and after UV treatments, even in low-risk patients. Additionally, clinicians should consider spacing light treatments more cautiously and maintain a low threshold for deferring further adjustments in patients with even subtle macular changes. Finally, as in all case reports, these findings should be interpreted with appropriate caution. Although definitive causality cannot be established, the symmetry, timing, and favorable response to conservative therapy make this a clinically meaningful association worth further exploration. 

## Conclusions

The LAL is an excellent tool in the area of refractive cataract surgery. However, UV exposure associated with LAL treatments may be associated with CME in the postoperative period. Decreased BCVA secondary to CME can be reversed with early intervention involving topical NSAID and prednisone in eyes with no previously underlying retinal pathology. We recommend that further UV exposure be halted until complete resolution of CME has occurred. Secondly, we recommend prophylactic continuation of topical NSAID and prednisone until one month after final UV treatment in order to prevent recurrence of macular edema in these susceptible patients.
